# Host preference patterns in domestic and wild settings: Insights into *Anopheles* feeding behavior

**DOI:** 10.1111/eva.13693

**Published:** 2024-05-31

**Authors:** Lemonde Bouafou, Boris K. Makanga, Nil Rahola, Marilou Boddé, Marc F. Ngangué, Josquin Daron, Audric Berger, Theo Mouillaud, Alex Makunin, Petra Korlević, Joachim Nwezeobi, Pierre Kengne, Christophe Paupy, Mara K. N. Lawniczak, Diego Ayala

**Affiliations:** ^1^ UMR MIVEGEC, University of Montpellier, CNRS, IRD Montpellier France; ^2^ CIRMF Franceville Gabon; ^3^ IRET Libreville Gabon; ^4^ Wellcome Sanger Institute Hinxton UK; ^5^ ANPN Libreville Gabon; ^6^ Medical Entomology Unit Institut Pasteur de Madagascar Antananarivo Madagascar

**Keywords:** *Anopheles*, Gabon, host preference, malaria, phenotypic plasticity, protected areas

## Abstract

The adaptation of *Anopheles* malaria vectors to domestic settings is directly linked to their ability to feed on humans. The strength of this species–habitat association is unequal across the species within the genus, with the major vectors being particularly dependent on humans. However, our understanding of how blood‐feeding behavior interacts with and adapts to environmental settings, including the presence of humans, remains limited. Using a field‐based approach, we first investigated *Anopheles* community structure and feeding behavior patterns in domestic and sylvatic settings in La Lopé National Park in Gabon, Central Africa. We characterized the preference indices using a dual‐host choice sampling approach across mosquito species, habitats, and seasons. We then quantified the plastic biting behavior of mosquito species in each habitat. We collected individuals from 16 *Anopheles* species that exhibited significant differences in species composition and abundance between sylvatic and domestic settings. The host‐seeking behavior also varied among the seven most abundant species. The general attractiveness to each host, human or animal, remained relatively constant for each species, but with significant variations between habitats across species. These variations, to more generalist and to more anthropophilic behavior, were related to seasonal changes and distance from the village, respectively. Finally, we pointed out that the host choice of major malaria vectors changed in the absence of humans, revealing a plastic feeding behavior of these species. This study highlights the effect of humans on *Anopheles* distribution and feeding evolution. The characterization of feeding behavior in wild and domestic settings provides opportunities to better understand the interplay between genetic determinants of host preference and ecological factors. Our findings suggest that protected areas may offer alternative thriving conditions to major malaria vectors.

## 1 INTRODUCTION

Before humans extensively populated Africa, thus modifying and transforming the landscape of this continent, *Anopheles* mosquitoes likely thrived in natural settings and fed on animals. When humans were just a sporadic and minor blood‐meal source (60–40 Kya), malaria parasites were likely transferred from great apes to humans in the forests of Africa (Otto et al., 2018). The potential bridge vectors may have included the sylvatic species *Anopheles moucheti* and *Anopheles vinckei*, which exhibit a generalist host preference (Makanga et al., 2016; Paupy et al., 2013). The advent of agriculture changed the course of malaria history (Tishkoff et al., 2001). Human settlements and population densities greatly increased and spread across the continent during the last 8–6 Kya (Barker & Goucher, 2015), leading to habitat transformations and creating new ecological settings. Several mosquito species adapted to domestic settings benefiting from their close association with humans, from whom they acquired all the resources they needed for their survival and development (Mouchet et al., 2004; Robert et al., 2017). Then, by direct (e.g., fitness) or indirect (e.g., larval breeding) selection pressures, these mosquitoes transitioned from a sylvatic to a domestic ecology. They thus specialized in feeding on humans, resulting in the epidemiological consequence of transmitting malaria parasites to human populations in Africa.

The evolution of host preference, from nonhuman animals to humans, has not been homogeneous across the anopheline community (Besansky et al., 2004; Takken & Verhulst, 2013). Several species rapidly adapted to domestic settings, exhibiting pronounced preference for humans, while others remained zoophilic, feeding on animals (Carnevale et al., 2009). Host preference is modulated by various intrinsic and extrinsic factors, including host availability, physiological state, and genetic background (Takken & Verhulst, 2013). Moreover, in malaria mosquitoes, the proportion of human blood meals can vary both spatially and temporally within the same species. For instance, population of *Anopheles arabiensis* exhibits a higher degree of anthropophilic behavior in West Africa than in Madagascar (Duchemin et al., 2001). The same mosquito species displays high zoophilic behavior in Tanzania during the wet season (Katusi et al., 2022). Importantly, even the most human‐specialized mosquitoes can feed on multiple hosts. Major malaria vectors have been recurrently found feeding on other animals (Takken & Verhulst, 2013). Moreover, the trophic behavior can quickly vary in response to environmental pressures. For instance, the extensive use of insecticide‐treated nets might increase the proportion of mosquitoes feeding on animals (Gatton et al., 2013; Saul, 2003).

In the last century, especially in the latter half, most research focused on highly anthropophilic mosquitoes due to their significant role in malaria transmission (Mouchet et al., 2004). Indeed, while major malaria vectors have been recorded in thousands of villages across sub‐Saharan Africa (Kyalo et al., 2017), to date, they have never been studied in the absence of humans, assuming that they can only thrive in domestic conditions. Therefore, wild areas, such as natural parks and other protected habitats, have been often not included in malaria studies. Consequently, it is not well‐known how *Anopheles* specialized on humans. In *Aedes aegypti*, researches have unveiled the mechanisms and dated the evolution of the anthropophilic feeding behavior, thanks to the co‐occurrence of domestic and sylvatic populations (Powell & Tabachnick, 2013; Rose et al., 2020, 2023). In the last years, several studies incidentally reported the presence of major malaria vector species in natural parks in Gabon (Barron et al., 2019; Makanga et al., 2016; Paupy et al., 2013), Madagascar (Zohdy et al., 2016), and South Africa (Munhenga et al., 2014), suggesting that sylvatic areas could host wild populations of those species, possibly in many natural areas of Africa. The presence of such malaria vectors within natural parks might help to understand the specialization of *Anopheles* on humans. Moreover, they could alter the epidemiological balance by facilitating zoonotic pathogens transfer between wildlife and human compartments (Obame‐Nkoghe et al., 2023; Xia et al., 2020). Whether these vectors could also exploit parks as a refuge against conventional vector control strategies (i.e., indoor insecticide spray) remains unknown. This is a new evolutionary scenario, not yet considered in malaria control programs.

In this study, we investigated the host‐seeking behavior of sylvatic and domestic populations of *Anopheles* mosquitoes, including the major vectors *An. coluzzii, An. gambiae*, and *An. funestus*, in La Lopé National Park, Gabon, Central Africa. Our field‐based approach and statistical models allowed us to delve into the host preference in *Anopheles* species according to habitats and seasons. We observed a stable presence of major malaria mosquitoes away from any human activity. Moreover, these major malaria mosquitoes displayed a marked preference for humans also inside the protected area, though their feeding behavior might change from anthropophilic to zoophilic in function of the host availability. Conversely, most secondary malaria vectors exhibited a zoophilic or generalist host preference, but increased their anthropophilic behavior with the distance from domestic settings. The presence of major malaria vectors inside natural parks and their feeding on animals raises new questions about the role of national parks in malaria transmission (Durrheim et al., 1998).

## 2 MATERIALS AND METHODS

### 2.1 Research permits and ethical approval

The mosquito collections and the methods used in this study were approved by the Gabon National Research Council (research permit PROT N°016/2019/PR/SG/CNE), including the human landing catch (HLC) method, following the recommendations of the National Ethical Committee for the Research in Gabon (ethical clearance N°0031/2014/SG/CNE). Moreover, an access permit was granted to enter and work in the National Park of La Lopé (entry authorization AR011/21/MESRSTT/CENAREST/CG/CST/CSAR).

### 2.2 Description of the study sites

**FIGURE 1:**
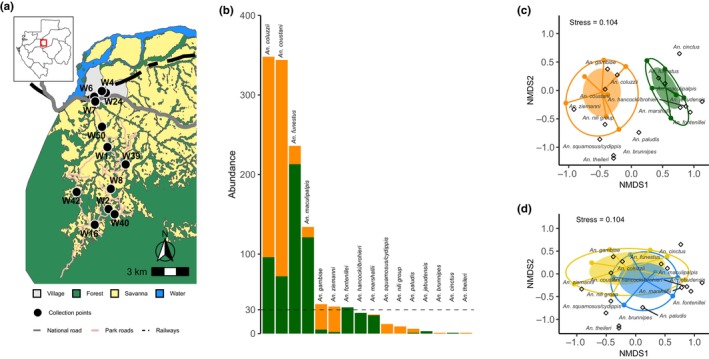
Sampling sites and overview of the *Anopheles* community. (a) Map of the sampled sites in the village (domestic) and in the National Park (sylvatic) of La Lopé. (b) Histogram showing the number of individuals of the different *Anopheles* species collected in the two habitats (green = sylvatic, orange = domestic). An arbitrary number of 30 individuals (dashed horizontal line) was established as the threshold to select species for the host preference analysis. Nonmetric multidimensional scaling (NMDS) was used to determine the mosquito communities in the two habitats (c) and between the two seasons (d). Inner ellipses (fully colored) represent standard errors of the centroids, while the outer ellipse hulls enclose all the environmental sampling units (collection sites –C– and days of the season –D–). Species locations (coordinates) are represented by diamond shapes. Colors: green = sylvatic, orange = domestic, blue = rain season, yellow = dry season; Stress is a proportional measure of badness of fit. Stress value higher than 0.3 is considered as a bad representation.

La Lopé National Park is located in the central part of Gabon. It covers an area of 4913 km^2^, combining rainforest and a small part (10%) of savannah areas (Figure 1a). In the park, the weather is characterized by a long rainy season (from October to May) followed by a dry season (from June to September) with only some sporadic rains. A considerable interannual variation in the rainfall amount and distribution is observed. The forest–savanna mosaic in the northern part of the park is of great interest in terms of biodiversity and ancient human activities (Cuni‐Sanchez et al., 2016; Oslisly & White, 2000).

### 2.3 Mosquito collection and feeding preference assays

Mosquitoes were collected in the village of La Lopé (four domestic sites) and in the park (eight sylvatic sites, located away from any human presence or activity) (Figure 1a). Sampling was carried out using the HLC and animal‐bait catch (ABC) methods (Service, 1993; Silver & Service, 2008). Briefly, ABC consists of an untreated square mosquito net (3 m on each side) hanging 30 cm from the ground and tied to a metal frame. A 20‐cm inner eave tops the net to prevent mosquitoes from escaping during collection (Davidson et al., 2019). An adult sheep (*Ovis aries*), used as animal proxy, is tied in the center of the setup. Each sheep was dewormed, and when deployed inside the National Park, a sheet was used to recover all its excrements in order to protect the local wildlife from any risk of infestation (protocol approved by the Gabon National Agency of National Parks). Mosquitoes were collected from 6.00 pm to midnight (6 h). Mosquitoes resting in the ABC net were individually aspirated using a mouth aspirator by two collectors every hour. Mosquitoes collected by HLC and ABC were killed in a freezer at −20°C. All specimens were then identified at the species or species complex/group level according to their morphological characteristics (Gillies & Coetzee, 1987; Gillies & de Meillon, 1968). All samples were individually preserved in 1.5 mL microcentrifuge tubes with desiccant (silica gel) and stored at −20°C for further analysis.

Two different experiments, referred to as “two‐choice” and “no‐choice,” were carried out to assess the host preference, in function of spatiotemporal variables and behavioral plasticity, respectively. In the “two‐choice” experiment, mosquitoes were collected in both domestic and sylvatic habitats and in two periods, at the end (April 2019) and at the beginning (November 2019) of the rainy season. Seasons might be characterized by little variation in human activities; on the other hand, differences in the distribution of animal hosts are expected. At each sampling site, a dual‐choice system was deployed. This consisted of a Latin square formed by two humans (HLC, anthropophilic behavior) and two animals (ABC, zoophilic behavior). This allowed mosquitoes to choose their preferred host while avoiding a position effect. The “no‐choice” experiment was carried out to evaluate the feeding behavior when only one host is available. In October 2021, mosquitoes were collected using only animals (ABC) in sylvatic settings and only humans in domestic settings.

### 2.4 Taxonomic and molecular species identification by targeted amplicon sequencing

Mosquitoes were first sorted by genus based on morphological characters. Then, only specimens belonging to the *Anopheles* genus were identified at the species level using dichotomous taxonomic keys (Coetzee, 2020; Gillies & Coetzee, 1987; Gillies & de Meillon, 1968). At La Lopé, three members of the *An. gambiae* complex (*An. gambiae*, *An. coluzzii*, and *An. fontenillei*) were previously reported (Barron et al., 2019). Therefore, mosquitoes belonging to the *An. gambiae* complex were processed for molecular analysis. Total genomic DNA was extracted from head–thorax using the Qiagen DNeasy Blood & Tissue Kit following the manufacturer's protocol (Qiagen). Then, genomic DNA samples were sent to Wellcome Sanger Institute (UK) for species identification using a recently developed targeted amplicon sequencing panel for *Anopheles* (ANOSPP) (Makunin et al., 2021). Briefly, genomic DNA was amplified using a multilocus amplicon panel that targets 60 loci in the nuclear genome of anopheline mosquitoes. A two‐stage multiplex PCR was performed before sequencing using Illumina Miseq (Makunin et al., 2021). Species assignment was then performed using the NNoVAE method, a *k‐*mer‐based distance method that consists of a first‐step prediction using the nearest neighbor approach followed by a variational autoencoder step (Boddé et al., 2022). Besides *An. gambiae* mosquitoes, a subset of 360 randomly selected *An. funestus* specimens also was analyzed using the ANOSPP.

### 2.5 Blood meal identification

Host DNA of blood‐engorged mosquitoes collected in each habitat was used to determine the origin (human vs. sheep) of the blood meals. A 360‐bp fragment of the cytochrome oxidase subunit I (*COI*) gene was amplified using a set of primers [COI_short (f): GCAGGAACAGGWTGAACCG; COI_short (r): AATCAGAAYAGGTGTTGGTATAG] designed by Townzen et al. (2008). PCR amplification was performed using a 50‐μL reaction mixture that contained 5 μL of 10X reaction buffer (Qiagen), 5 μL of 25 mmol/L MgCl_2_, 2 μL of 10 μmol/L of each primer, 2.5 μL of 20 μmol/L dNTPs, 0.2 μL of Taq DNA polymerase (Eurogentec, Belgium), and 3 μL of template DNA. The following PCR program was used: initial denaturation at 95°C for 3 min followed by 35 cycles at 95°C for 30 s, 47°C for 50 s, 72°C for 1 min, and final extension at 72°C for 5 min. After checking the expected size and the quality of the signal on 1.5% agarose gel, PCR products were sent to Eurofins Genomics for sequencing. Sequence editing and alignment were done using Geneious Prime (Biomatters). The identity of vertebrate blood was inferred by looking for homologous host sequences in GenBank (https://blast.ncbi.nlm.nih.gov/).

### 2.6 Statistical analyses and data visualization

How habitat affects the mosquito community structure was visually assessed by performing a nonmetric multidimensional scaling (NMDS) analysis using the package *vegan* (function *metaMDS*) (Oksanen et al., 2022). Data on species composition from the “two‐choice” experiment were aggregated for each collection night and site. Differences in species communities between habitats were tested by fitting a negative binomial model for multivariate abundance data using the package *mvabund*, function *manyglm* (Wang et al., 2012). Then, the indicator species analysis was performed to assess species–habitat associations. The indicator species analysis allows us to assess the ecological niche preferences of the different *Anopheles* species by analyzing abundance data in each habitat type. This analysis was carried out with the *indicspecies* package, including the “*r.g*” argument for the species–site group association function, to correct for unequal group sizes (Cáceres & Legendre, 2009; De Cáceres et al., 2010). This analysis allows determining lists of species associated with a given habitat.

To study the host preference behavior of mosquitoes, different generalized linear mixed models (GLMM) were fitted with the *glmmTMB* package (Brooks et al., 2017). To analyze the host choice preference (coded as binary response, animal = 0; human = 1) in function of the habitat and season, the species, habitat type, and season variables were included in the model as fixed effects (with an interaction between species and season), while the collection day and collection sites were used as random effects. For data visualization, the probabilities (*p*) were estimated and then transformed into preference index (PI) using the formula PI = 2*p* − 1 (McBride et al., 2014; Rose et al., 2020). The PI values ranged from −1 to 1; PI = 0 means that mosquitoes showed an equal likelihood of choosing either host (no preference). A PI above or below zero means that the mosquitoes were more likely to choose humans or sheep, respectively. Probability estimations and post hoc analyses, and pairwise comparisons of estimated marginal means (EMMs) were performed with the function *emmeans* (package *emmeans*) (Lenth, 2023). A similar model, as previously described, was fitted to understand host preference pattern changes along a geographical gradient, from the village to the forest. Predictors were species, season, and collection site. We defined an interaction between species and season as it significantly improved model fit. The day of collection was used as a random variable. PI values were derived from the model, and correlation tests were conducted against geographical distance. Geographical distance was estimated by replacing each categorical site with its respective distance value to the village, calculated using the package *geosphere* (Hijmans, 2022). To assess the relationship between PI and distance, we employed the Pearson's correlation test when the data met its assumptions. Otherwise, we used the Spearman's rank correlation test.

The mosquito response in the presence of one or two hosts in each habitat was assessed by fitting generalized Poisson and Conway‐Maxwell Poisson regression models for each domestic and sylvatic habitat, respectively. The numbers of mosquitoes collected on each host for each habitat (i.e., humans in domestic vs. animals in sylvatic settings) were compared when the host was alone or together with the alternative host. The log of the overall count of mosquito collected by day for each species was included as offset in the models. Model selection was based on a likelihood ratio test. The goodness of fit for each model was assessed by visually inspecting and testing the simulated residuals with the *DHARMa* package (Hartig, 2022). All statistical analyses and data visualization were performed with R version 4.2.1 (R Core Team, 2022). Data were visualized with the package *ggplot2* (Wickham, 2016) and companions packages, such as *circlize* (Gu et al., 2014) and *ggordiplots* (Quensen, 2018). All reported *p* values are two tailed.

## 3 RESULTS

### 3.1 Anopheline community diverges between natural and domestic habitats

In total, 2670 mosquitoes were collected from sylvatic and domestic sites (Figure 1a) during this study: 2180 from “two‐choice” and 490 from “no‐choice” experiments (Table S1). The collected mosquitoes belonged to five genera: *Anopheles* = 1713 (64.16%), *Aedes* = 502 (18.8%), *Culex* = 277 (10.37%), *Mansonia* = 172 (6.44%), and *Coquillettidia* = 6 (0.22%) (Figure S1 and Table S1). A total of 16 *Anopheles* species were identified with different vectorial importance (Table S2), and the most abundant were *An. coluzzii* (*n* = 408), *An. coustani* (*n* = 356), *An. funestus* (*n* = 289), and *An. maculipalpis* (*n* = 174) (Table S2). Few species were not possible to assign them to a unique taxon. For instance, we opted to keep *An. hancocki/An. brohieri* because at La Lopé they are morphologically indistinguishable due to both presenting all‐white tarsomeres III‐3 to III‐5 and wing characteristics (Coetzee, 2020; Gillies & Coetzee, 1987; Gillies & de Meillon, 1968). Similarly, we decided to combine *An. squamosus/An. cydippis* because they can only be differentiated at the egg or larval stage.

To investigate the *Anopheles* community structure, the “two‐choice” (HLC and ABC) collections were used because the sampling design and effort were consistent across habitats and allowed gathering a wider diversity of mosquitoes (zoophilic and anthropophilic vectors). Unidentified (morphologically and/or molecularly) were excluded from the subsequent analyses (*n* = 218 mosquitoes). Therefore, 1249 anopheline mosquitoes were used for the community structure analyses (Figure 1b). Our statistical analyses found significant differences between sylvatic and domestic habitats in terms of species composition and abundance (Figure 1b,c), confirming that the habitat type is an ecological driver of community composition at this fine geographical scale (multivariate GLM, Deviance = 79.13, *p* = 0.003). There was substantial variability in species–habitat associations (Figure 1b,c). For instance, no *Anopheles* species was significantly associated with domestic settings, including the major malaria mosquitoes *An. coluzzii* (indicator species analysis, *r*_pb_ = 0.366, *p* = 0.224) and *An. gambiae* (*r*_pb_ = 0.559, *p* = 0.0605). Conversely, the other major mosquito species collected in our study, *An. funestus*, exhibited a strong species–habitat association but in sylvatic settings (*r*_bp_ = 0.883, *p* < 0.001). Other species, such as *An. maculipalpis* (*r*_bp_ = 0.709, *p* = 0.003), *An. fontenillei* (*r*_pb_ = 0.573, *p* = 0.021), and *An. hancocki/brohieri* (*r*_pb_ = 0.570, *p* = 0.0213) were associated with the sylvatic habitat. Neither the season (Figure 1d) nor the habitat–season interaction had a significant impact on the community structure (multivariate GLM, Deviance = 35.13, *p =* 0.080; Deviance = 18.37, *p* = 0.086, respectively). Therefore, community structure was mainly driven by the strong association of *Anopheles* species with sylvatic rather than with domestic areas.

### 3.2 Spatial and seasonal host‐feeding changes in *Anopheles* species

**FIGURE 2:**
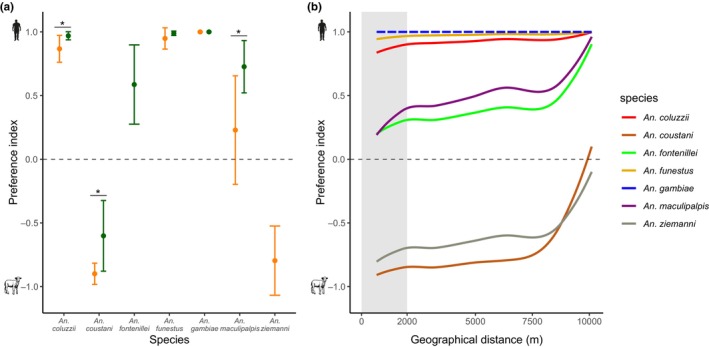
*Anopheles* host preference in domestic and sylvatic habitats. (a) Comparison of the host preference for the same *Anopheles* species in domestic and sylvatic habitats. Dots are the mean preference index ± 95% CIs. Feeding behavior was analyzed by fitting a GLMM, and *post hoc* comparisons were performed using the *emmeans* package. Then, for data visualization, we calculated from the model the preference index from the estimated probability for a mosquito to feed on a human host (see Section 2 for details), and only considered species with ≥5 individuals per habitat. (b) Preference index variations along the geographical gradient from the village (gray shading) to inside the park. The preference index was calculated from a GLMM fitted beforehand. The correlation between preference index and distance was tested using Pearson's and Spearman's correlation tests. Dashed line indicates not significant correlation, while full line means significant relationship between preference index and distance.

To explore how mosquitoes adjust their feeding behavior to the presence of different hosts, “two‐choice” experiment were carried in both domestic (village) and sylvatic (park) settings. For statistical power purposes, a threshold of 30 individuals per *Anopheles* species was arbitrarily established (Figure 1b). Consequently, only seven species were retained for the subsequent analysis. Spatial host preference differences were examined for each of these seven *Anopheles* species. First, the PI in each habitat (i.e., domestic and sylvatic) was estimated for the seven *Anopheles* species. Our results allowed differentiating anthropophilic (human choice) and zoophilic (animal choice) mosquitoes (Figure 2a). Most *Anopheles* species found in the two habitats exhibited a similar trophic behavior across habitats. Sylvatic and domestic populations of *An. coluzzii*, *An. funestus*, and *An. gambiae* displayed a strong anthropophilic behavior (Figure 2a) (PI_domestic_ = 0.87, PI_sylvatic_ = 0.97, *p* = 0.034; PI_domestic_ = 0.95, PI_sylvatic_ = 0.99, *p* = 0.258; PI_domestic_ = 1, PI_sylvatic_ = 1, *p* = 0.985, respectively). Conversely, *An. coustani* and *An. ziemanni* (PI_domestic_ = −0.9, PI_sylvatic_ = −0.6, *p =* 0.026; PI_domestic_ = −0.8, respectively) displayed a zoophilic behavior. All species apart from *An. gambiae* and *An. funestus* were more attracted to humans in the sylvatic habitat, even species associated with sylvatic settings, such as *An. maculipalpis* or *An. fontenillei* (PI_*domestic*_ = 0.23, PI_*sylvatic*_ = 0.73, *p* = 0.023; PI_sylvatic_ = 0.59, respectively). To better understand this pattern, the PI for each collected site, from the village to the more distant site in the forest, was estimated. The hypothesis was that the zoophilic behavior should increase with the distance from the village. However, all mosquito species, aside from *An. gambiae* that showed a consistent trend, exhibited a positive correlation between the PI and geographical distance (Figure 2b and Table S3). In other words, the anthropophilic feeding behavior of mosquitoes significantly increased with the distance away from the village (Figure 2b).

**FIGURE 3:**
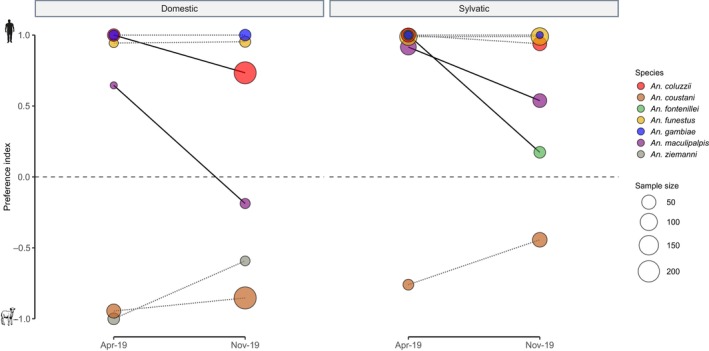
Seasonal host preference variations. Dashed lines indicate no significant variation in feeding preference (pairwise comparisons of EMMs, *p* > 0.05) between seasons, while full lines indicate significant change for a given species (pairwise comparisons of EMMs, *p* < 0.05). Post hoc comparisons were carried out when ≥5 individuals per species were collected in each habitat and in each season.

Host availability can vary spatially and temporally. To better understand this interaction, in each habitat, the feeding patterns (PI) were compared between collection periods (Figure 3). *Anopheles gambiae* and *An. funestus* did not display any statistical variation in PI between the two seasons. The anthropophilic behavior of both species was stable between seasons, regardless of habitat type (pairwise comparisons of EMMs, *p* > 0.05). Conversely, the other mosquito vectors showed a tendency toward a generalist behavior at the beginning of the rainy season (November 2019). Nonetheless, this feeding change was only significant for more anthropophilic species when analyzed individually. Indeed, *An. coluzzii*, *An. fontenillei*, and *An. maculipalpis* exhibited a significant decrease in their degree of anthropophily between April and November (pairwise comparisons of EMMs, *p* < 0.05; Table S4). Despite the increasing trend in the PI, it was not statistically significant for *An. coustani* and *An. ziemanni* (Table S4).

During the study, 15 blood‐fed mosquitoes were screened. The blood sequences perfectly matched with the host used in each method (sheep in ABC and human in HLC) (Table S1). This result confirmed the limited risk of misassignment individuals between methods.

### 3.3 Major malaria vectors exhibit host preference plasticity in the absence of humans

**FIGURE 4:**
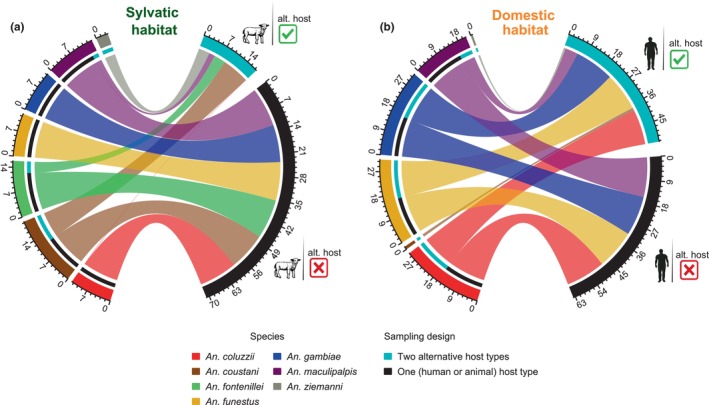
Mosquito feeding behavior in two‐choice and no‐choice sampling design. Chord diagrams showing the number of *Anopheles* mosquitoes collected when given the choice to feed on two alternative hosts (human vs. sheep) compared to a no‐choice situation (only one host available). While the two‐choice experiment aimed to assess the feeding preference of mosquito species, the no‐choice sampling design aimed to estimate the potential feeding shift according to host availability. It consisted of using sheep under sylvatic and human under domestic settings, in order to mimic natural host availability. (a) Number of mosquitoes collected only from animals in the two‐choice versus no‐choice sampling design in sylvatic settings. (b) Number of mosquitoes collected only from humans in the two‐choice versus no‐choice sampling design in domestic settings. Green tick and red cross‐checkmarks indicate the presence (two‐choice) or absence (no‐choice), respectively, of an alternative host type in the sampling design. Outer tracks or sectors represent the total number of mosquitoes for each species, and for each collection method. The inner track represents, for a given species, the proportion of mosquitoes collected in each sampling design. Mosquito counts were estimated from the Poisson‐type GLMM fitted for each habitat.

While zoophilic mosquitoes could persist in the village feeding on domestic animals, the question arises for how anthropophilic species could feed under sylvatic conditions, specifically in the absence of humans. Phenotypic plasticity, referred to as the ability of a mosquito to adapt its host preference in function of the host availability, was assessed using the “no‐choice” collection method: the animal host (ABC) in sylvatic settings and the human host (HLC) in domestic settings. Figure 4 presents the results of our modeling approach to estimate the numbers of mosquitoes collected when a single host, no‐choice (human or sheep), and when both hosts, choice (human and sheep), were available. When given the choice of feeding host (i.e., “two‐choice” experiment), the three major malaria vectors collected in the current study (*An. coluzzii*, *An. funestus*, and *An. gambiae*) showed a strong preference for humans in sylvatic settings (Figures 2a and 3). However, with the “no‐choice” experiment, a significant shift in the feeding behavior of these three species was observed (Figure 4a). Indeed, in the absence of their preferred host, on average, 12.45 (GLMM, difference between the estimated daily counts from “no‐choice” and “two‐choice” experiments, z.ratio = 4.3, *p* < 0.001), 12.69 (z.ratio = 4.61, *p* < 0.001), and 12.35 (z.ratio = 2.17, *p* = 0.03) more mosquitoes were collected daily on animals, compared with the “two‐choice” experiment, for *An. coluzzii*, *An. funestus*, and *An. gambiae*, respectively. It revealed an elevated degree of host‐feeding plasticity from humans to animals. Like for the three major malaria vectors, the estimated number of mosquitoes collected on animals also significantly increased for other *Anopheles* mosquitoes that displayed relevant anthropophilic behavior in the sylvatic habitat (Figure 2a): *An. maculipalpis* (+11, z.ratio = 4.33, *p* < 0.001) and *An. fontenillei* (+9.23, z.ratio = 2.83, *p* = 0.005) (Figure 4a). On the other hand, the estimated number of collected zoophilic *An. coustani* mosquitoes did not significantly change in the absence or presence of human hosts (+3.41, z.ratio = 1.07, *p* = 0.28) (Figure 4a).

The same assay was carried out in the village (domestic settings) using as “no‐choice” the human host. In the absence of the animal proxy, the attractiveness toward humans of the anthropophilic species *An. coluzzii* (+1.66, z.ratio = 1.14, *p* = 0.254), *An. gambiae* (−0.044, z.ratio = −0.018, *p* = 0.986), and *An. funestus* (+0.58, z.ratio = 0.239, *p =* 0.811) did not change (Figure 4b). However, no *An. coustani* and *An. ziemanni* were collected on humans in the domestic habitat when the animal host was absent. This suggests that such mosquitoes are not attracted to humans, and might look for other animals.

## 4 DISCUSSION

The distribution of anopheline mosquitoes and their behavioral traits (e.g., blood‐feeding behavior) can be affected by ecological features and their evolutionary trajectory (Kamdem et al., 2012; Moiroux et al., 2012; Neafsey et al., 2015; Small et al., 2020). In this study, we analyzed and compared the community structure and blood feeding preference of *Anopheles* mosquitoes collected in domestic and sylvatic settings. Our results show a significant effect of the human presence on the anopheline biodiversity and abundance, and revealed the presence of major malaria vectors within the natural park protected areas (Figure 1). The host‐feeding preference remained stable between habitats, but a general human preference increase was observed for all species in sylvatic settings (Figure 2). Despite the genetic determinants of host preference, mosquitoes can vary their feeding preference according to the ecological settings. We observed a more opportunistic behavior at the beginning of the rainy season (Figure 3). Moreover, host availability and mosquito density can mediate host selection. Based on our ecological modeling approach, we observed a shift on host feeding in the absence of the preferred host (Figure 4), explaining how highly anthropophilic mosquitoes can persist in sylvatic settings in the absence of humans. Although these conclusions are limited to the National Park of La Lopé, they open new perspectives about the impact of protected areas on malaria control.

The specialization of mosquitoes for domestic settings, which offer abundant human prey and opportunities for larval breeding and adult resting, has drastically influenced the course of human history (Powell & Tabachnick, 2013; White et al., 2011). Our study confirmed that even at a very short spatial scale (~15 km) and under the same ecological conditions, domestic and sylvatic settings exhibit distinct species composition and abundances (Figure 1b,c). In mosquitoes, anthropogenic activities can contribute to the segregation of mosquito communities, as observed in other protected areas of Africa (i.e., Kruger Park) (Schrama et al., 2020). At La Lopé village, the human impact on land use is very limited due to the absence of extensive farming or ranching activities. Therefore, the mosquito distribution is mainly determined by breeding site availability and flight capacity. Among the 16 *Anopheles* species collected at La Lopé, half were found in both habitats (Figure 1b). This result suggests that most *Anopheles* species, including both major and secondary malaria vectors, could breed and/or freely circulate in the village and the park, leading to epidemiological consequences for pathogen transfer (Kraemer et al., 2019; Makanga et al., 2016; Obame‐Nkoghe et al., 2023). The presence of malaria vectors in protected areas of Africa has been already sporadically reported (Barron et al., 2019; Costantini & Diallo, 2001; Munhenga et al., 2014; Paupy et al., 2013; Zohdy et al., 2016), indicating that mosquitoes might have retained their ancestral ability to thrive in natural sites. Interestingly, the major malaria vector *An. funestus*, known for its high degree of anthropophily and endophily (Dia et al., 2013), was significantly associated with sylvatic settings (Figure 1b). This is likely related to their larval and/or adult ecology. The sylvatic area provides several larval habitats characterized by larger and more permanent or semipermanent water bodies containing aquatic vegetation and algae, making them suitable for species like *An. funestus* (Gimnig et al., 2001; Nambunga et al., 2020). In addition to Gabon (Paupy et al., 2013), this mosquito has been reported in sylvatic areas across Africa, including Uganda (Hamon, 1955) and Kenya (Eastwood et al., 2020). Its extraordinary flight capacity (up to 7 km) could explain the distance between breeding and feeding sites (Dia et al., 2013; Hamon, 1955).

In *Anopheles*, the preference for feeding on humans is more an exception than the norm (Besansky et al., 2004; Clements, 1999). In this study, we examined how the presence of humans affects their feeding behavior. Despite the significant differences in anopheline community structure between habitats, we observed a constant feeding preference pattern across species and habitats (Figure 2a). In general, major malaria vectors remained highly anthropophilic, while the secondary vectors or nonvectors exhibited a more zoophilic or generalist feeding behavior. Nonetheless, females of several populations significantly differed in host preference from the domestic (village) to the sylvatic areas (Figure 2a). In mosquitoes, host preference is a trait modulated by specific genes (Main et al., 2016; McBride et al., 2014; Neafsey et al., 2015), and it can be rapidly selected after few generations (Gillies, 1964; Takken & Verhulst, 2013). Therefore, we expected anthropophilic behavior in the domestic sampling sites (village) and zoophilic, or generalist, behavior in the sylvatic sites. The close proximity between domestic and sylvatic sites (up to 15 km, Figure 1a) suggests that mosquitoes are unstructured, thus forming a panmictic population (Taylor et al., 2001). Therefore, gene flow could disrupt habitat‐feeding specialization. Similar findings were reported for *Ae. aegypti*, the yellow fever mosquito. In Gabon and Kenya, domestic and sylvatic *Ae. aegypti* populations are genetically connected and show a similar human host preference (Kotsakiozi et al., 2018; Rose et al., 2020; Xia et al., 2020). Noteworthy, the major malaria vectors *An. gambiae, An. coluzzii*, and *An. funestus* remained highly anthropophilic in sylvatic settings. Costantini and Diallo (2001) already showed in a similar ecological scenario that forest populations of *An. gambiae* and *An. funestus* in Senegal are more attracted to humans than to monkeys (C Costantini & Diallo, 2001). The higher fitness by feeding on human hosts in these species might explain their human preference (de Swart et al., 2023). However, a recent study in Burkina Faso showed no fitness cost for feeding on alternative domestic hosts (cow and sheep), compared with humans, in *An. coluzzii* (Vantaux et al., 2023). Therefore, the strong association for feeding on humans should involve other biological determinants such as a greater sensitivity of anthropophilic mosquitoes to detect human skin volatiles. Host seeking and selection in mosquitoes is the product of a complex process that can be shaped at short range by a variety of olfactory and visual stimuli (Cardé, 2015). The difference in composition and proportion of olfactory cues produced by the different human and animal hosts could affect mosquito host selection (McBride et al., 2014). In addition to odorant compounds, physical factors including heat and humidity, and visual factors such as the color of sheep coats or the clothing colors of collectors could play a role in host selection by the different *Anopheles* species, particularly in sylvatic environments where this contrast would be more pronounced (Alonso San Alberto et al., 2022; Hawkes et al., 2017; Van Breugel et al., 2015). Other biological determinants such as learning and physiological status could also have a significant effect on the trophic behavior of these mosquito vectors (Takken & Verhulst, 2013).

Interestingly, *An. fontenillei*, the recently discovered species within the *An. gambiae* complex (Barron et al., 2019; Paupy et al., 2013), strongly preferred humans, though it has never been found in domestic settings (Figures 1b, 2a and Table S1). Anthropophilic behavior is a common trait within the complex (White et al., 2011). Indeed, other wild‐living and zoophilic species also display high preference for humans in natural conditions (Akogbeto & Romano, 1999; Makunin et al., 2021; Pates et al., 2001), including its closest sister species *Anopheles bwambae* (White, 1985). As Barron et al. (2019) observed signals of hybridization between *An. fontenillei* and *An. gambiae/An. coluzzii*, the extensive genetic exchange within the complex, particularly with the dominant and anthropophilic species, could explain the adaptative introgression of odorant receptors and gustatory receptors, maintaining a genetic polymorphism for feeding on humans (Fontaine et al., 2015; Neafsey et al., 2015). Other *Anopheles* species exhibited a preference for zoophilic or opportunistic feeding behavior, consistent with their secondary or negligible role in malaria transmission (Hamon & Mouchet, 1961).

We also estimated the PI variations across a spatial continuum between habitats. We hypothesized an increase of the animal preference with the distance from the village. However, our model revealed a significant and generalized increment of human preference, for anthropophilic and also zoophilic mosquitoes (Figure 2b). In mosquitoes, extrinsic factors, such as host accessibility and abundance, can determine host selection (Clements, 1999; Hamer et al., 2009; Takken & Verhulst, 2013). However, our “two‐choice” sampling method could be biased by the abundance of alternative prey in the nearby collection area. In other words, the collection of anthropophilic mosquitoes would be diluted by the number of other humans in the village. Conversely, in the forest, humans would more easily attract the anthropophilic proportion of the population due to the absence of human hosts. Therefore, the dilution effect relative to the most abundant prey reveals that feeding behavior can vary greatly in a very short distance. Interestingly, the preference variation was more prominent after 5–7 km from the village, more than the mosquito dispersion range, ~2.5 km, and therefore may limit gene flow and increase habitat specialization (Costantini et al., 1996). Overall, our results revealed that the behavioral preferences can change depending on the habitat type and host availability.

Behavioral plasticity is influenced by many extrinsic factors, such as host density and diversity (Ferraguti et al., 2013; Kent et al., 2009; Takken & Verhulst, 2013; Thiemann et al., 2011). At La Lopé, the strong seasonality in food resources (i.e., fruits, grass) affects the wildlife density and spatiotemporal distribution in the National Park (Tutin et al., 1991; White, 1994; White et al., 1995). Our seasonal analysis revealed a change in host preference across species. We observed a trend toward more generalist behavior in the rainy season (November) for all mosquito species, regardless of their overall feeding preference (Figure 3). This variation could be associated with rainfall levels and fruit abundance. Wild animals move freely between the village and the forest and they are more frequent during the fructification period and grass regrowth after fire management (Jeffery et al., 2014) (October to March) (Tutin et al., 1991; White et al., 1995). Moreover, during this period we observed the greatest abundance of mosquitoes due to rainfalls. This could facilitate genetic exchanges between domestic and sylvatic populations across species, homogenizing the feeding behavior. This result highlights the high plasticity of most of the studied *Anopheles* species, mediated by the host distribution and influenced by demographic events. This brings new questions concerning the importance of this plasticity and whether stable populations of highly anthropophilic mosquitoes can be maintained in sylvatic conditions. Our quantitative analysis using only the data from the “no‐choice” experiment in sylvatic conditions showed that mosquitoes can vary their choice when the preferred host is not available (Figure 4a). The average number of *An. coluzzii* and *An. funestus* increased by 12.45 and 12.69, respectively. Nonetheless, as genetic mechanisms determine host preference (McBride, 2016), we should assume a selection for more zoophilic or generalist mosquitoes that will be erased by extensive gene flow. Future population genetic studies should investigate the gene flow patterns and selection signatures between domestic and sylvatic populations. Moreover, although it would be challenging to handle wild animals of the park due to their protected status, it would be interesting to use these park‐inherent species as animal hosts to better assess the feeding behavior of mosquitoes inside the park.

## 5 CONCLUSION

*Anopheles* mosquitoes have evolved by adapting their behavior to their environment. In this study, we have highlighted the significant impact of human presence on mosquito species abundance and composition, even at a very small geographical scale. The feeding preferences remained relatively constant through species. However, they exhibited significant changes, associated with seasonality and host availability. Their ability to adapt their behavior allows them to colonize a wide range of habitats, including sylvatic settings. Genomic studies are needed to ascertain the population connectivity between habitats and whether this is an ancestral or novel adaptation process. Our results provide new evidence on the adaptive capacity of the main malaria vectors in Africa and suggest that they could use sylvatic areas as refuges in case of unsuitable conditions (i.e., vector control strategies) or competition for resources in villages. These findings could challenge malaria control efforts. However, these results cannot be generalized to all protected areas on the African continent. Further studies are therefore needed to understand the interaction between protected areas and malaria vectors across Africa.

## CONFLICT OF INTEREST STATEMENT

None declared.

## Supporting information


Figure S1.

Table S1.

## Data Availability

Additional supporting information can be found online in the Supporting Information section at the end of the article.
