# Supplementary figures and images for: Host preference patterns in domestic and wild settings: Insights into Anopheles feeding behavior
Source: Evol Appl. 2024 May 31;17(6):e13693. doi: 10.1111/eva.13693 (PMC11143308; doi:10.1111/eva.13693)

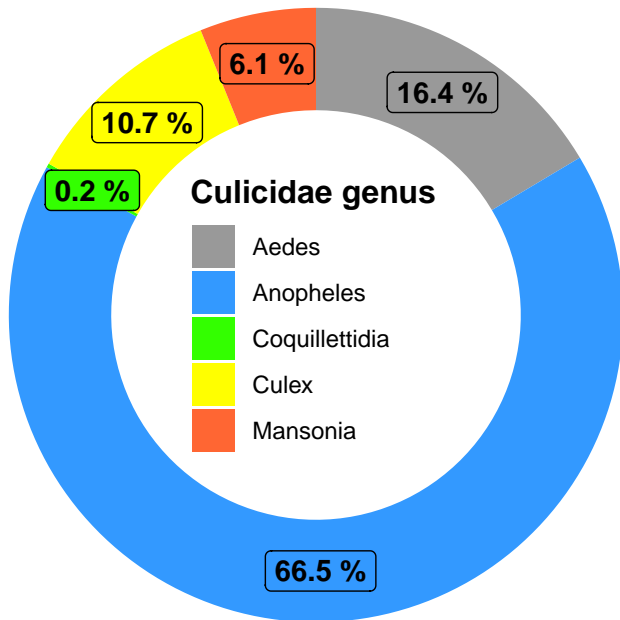

Supplement: Supplementary file 1 — Figure S1. [file EVA-17-e13693-s002.pdf]
